# A Low-Power Opamp-Less Second-Order Delta-Sigma Modulator for Bioelectrical Signals in 0.18 µm CMOS

**DOI:** 10.3390/s21196456

**Published:** 2021-09-27

**Authors:** Fernando Cardes, Nikhita Baladari, Jihyun Lee, Andreas Hierlemann

**Affiliations:** 1 Department of Biosystems Science and Engineering, ETH Zürich, 4058 Basel, Switzerland; nikhitabaladari@gmail.com (N.B.); jihyun.lee@bsse.ethz.ch (J.L.); andreas.hierlemann@bsse.ethz.ch (A.H.); 2 Section Bioelectronics, TU Delft, 2628 CD Delft, The Netherlands

**Keywords:** ADC, biosensor, CMOS, delta-sigma modulation, neural interface, VCO-ADC, VLSI

## Abstract

This article reports on a compact and low-power CMOS readout circuit for bioelectrical signals based on a second-order delta-sigma modulator. The converter uses a voltage-controlled, oscillator-based quantizer, achieving second-order noise shaping with a single opamp-less integrator and minimal analog circuitry. A prototype has been implemented using 0.18 μm CMOS technology and includes two different variants of the same modulator topology. The main modulator has been optimized for low-noise, neural-action-potential detection in the 300 Hz–6 kHz band, with an input-referred noise of 5.0 μV_rms_, and occupies an area of 0.0045 mm^2^. An alternative configuration features a larger input stage to reduce low-frequency noise, achieving 8.7 μV_rms_ in the 1 Hz–10 kHz band, and occupies an area of 0.006 mm^2^. The modulator is powered at 1.8 V with an estimated power consumption of 3.5 μW.

## 1. Introduction

Electrogenic cells, such as neurons, cardiac cells, or retinal cells, generate ionic currents across their membrane owing to the different ion channels that populate the cellular membranes. Transmembrane ionic currents produce voltage variations in the extracellular medium that can be detected by miniaturized sensors in close proximity to the cells. These voltage signals occur in different frequency bands and feature different amplitudes depending on their nature: neural action potentials (APs) manifest as spikes with amplitudes ranging from a few tens of μV to 1 mV and most signal power between 300 Hz and 6 kHz, whereas cardiac field potentials may reach tens of mV with most signal power in the 1 Hz–1 kHz band. The development of sensors, capable of simultaneously detecting action potentials of multiple cells, enables advanced electrophysiology studies, improving the understanding of complex signaling and opening paths to restoring lost functions. CMOS technology allows for low-noise recording from thousands of electrodes in parallel, either in vitro [1] or in vivo [2]. In vitro studies can be performed with CMOS microelectrode arrays (MEAs), the electrode array of which is co-integrated with the readout electronics [3]. In vivo interfaces commonly make use of passive probes connected to external readout arrays [2,4,5], although a variety of monolithic silicon probes combining electrodes and electronics on the same die have been developed [6,7].

In order to detect APs with acceptable signal-to-noise ratios, recording front-ends require input-referred noise values below 10 μV_rms_. This is especially relevant for neural interfaces, where small action potentials generated by different neurons need to be separated and assigned to the respective signal sources. CMOS technology allows for simultaneously recording from thousands of densely packed electrodes at a high spatiotemporal resolution, and it allows for conditioning and digitizing signals on-chip with low noise. Advanced signal post-processing (e.g., spike-sorting [8]) is frequently performed off-chip, due to the limited computing power of on-chip processors. This relieves the specifications of on-chip readout circuits, since some effects of circuit impairments, such as signal distortion, power-supply noise, and process variations, can be mitigated by means of calibration and post-processing. Furthermore, current trends in CMOS neural interfaces point towards low-power readout circuits, which enable the integration of tens of thousands of readout channels into the overall system without excessive power consumption and heat dissipation. A small footprint per channel is desired to minimize the area of the overall neural interface, which can easily exceed 100 mm^2^ [3,9].

Current state-of-the-art implementations of neural interfaces include a wide variety of analog-to-digital converter (ADC) topologies, such as successive approximation registers (SAR) [3,10,11,12], analog-to-time converters (ATC) [13], single-slope (SS) architectures [9,14], and different combinations of delta (∆) and delta-sigma (∆Σ) modulators [5,15,16,17]. ∆Σ modulators are well suited to low-frequency applications owing to noise-shaping, which reduces in-band quantization noise by means of high-pass filtering [18]. The in-band quantization noise depends on the sampling frequency and on different design parameters pertaining to the complexity of the modulator, such as the order of the high-pass filter and the number of bits of the quantizer. Voltage-control-oscillator (VCO)-quantizers have emerged as efficient implementations that provide one additional order of noise-shaping to the modulator [19,20]. This article presents a readout circuit for bioelectrical signals based on a continuous-time second-order ∆Σ modulator. Second-order noise-shaping is achieved by combining a single-loop first-order modulator with a 1-bit VCO-based quantizer. Analog circuitry has been simplified to reduce area and power consumption, while the circuit relies on off-chip digital post-processing for filtering, down-sampling, and further corrections. The prototype includes two variants of the same design: one design has been optimized for small real estate and action potential detection in the 300 Hz–6 kHz band, while the second design features a larger input stage to minimize the noise in the 1 Hz–10 kHz band.

This paper is organized as follows: In Section 2, the block diagrams of the proposed modulator and the circuits of the key building blocks are shown and described. The electrical characterization of the prototype is shown in Section 3, and the results of in vitro testing with cardiomyocytes are reported in Section 4. Finally, Section 5 concludes the paper.

## 2. Readout Design

**Figure 1:**
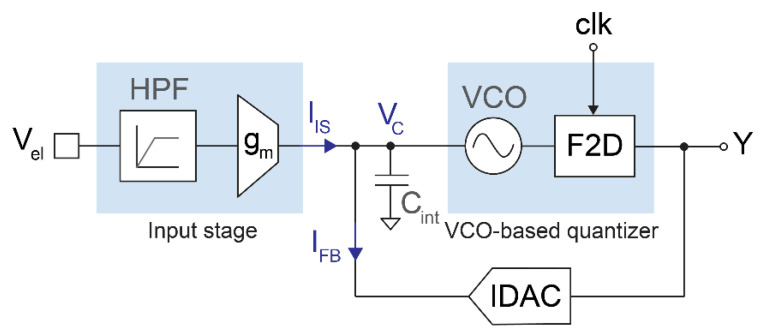
Simplified block diagram of the proposed opamp-less, second-order ∆Σ modulator with VCO-based quantizer.

Figure 1 shows a simplified block diagram of the proposed readout circuit. The input stage transforms the electrode voltage (V_el_) into a current (I_IS_) by means of a high-pass filter and an inverting transconductor. This current is injected into a capacitor (C_int_), which acts as the first integrator of the modulator. The capacitor voltage (V_C_) drives a VCO-based quantizer consisting of a VCO and a 1-bit frequency-to-digital converter, which is sampled at 1 MHz. The output bitstream drives the 1-bit current DAC that generates the feedback current (I_FB_) and closes the loop. The modulator resembles a first-order closed-loop ∆Σ architecture with an opamp-less integrator, using a VCO-based quantizer to achieve an additional order of noise shaping [19,21]. The sampling frequency is high enough to achieve an input-referred quantization noise below 2 μV_rms_ in the 300 Hz–6 kHz band with a 1-bit, second-order modulator, which keeps the structure of the converter very simple. Furthermore, the continuous-time topology obviates the need for an anti-aliasing filter due to the inherent low-pass filtering before sampling.

The modulator is stable, provided that the feedback current can rapidly counterbalance I_IS_ for any possible input signal so that the average current through C_int_ is kept at zero. In normal operation, the capacitor voltage V_C_ is an irregular triangular wave resembling the output of the first integrator of a continuous-time ∆Σ modulator. The average values of V_C_ and Y are defined by the transfer functions of the feedback I_DAC_ and the VCO-based quantizer. Under nominal conditions, ($\bar{I_{\mathrm{IS}}}$) = ($\bar{I_{\mathrm{FB}}}$) = 1.4 μA and ($\bar{V_{C}}$) = V_DD_/2 = 0.9 V, which sets the average VCO oscillation frequency close to f_s_/2 = 500 kHz and $\bar{Y}$ = 0.5.

### 2.1. Input Stage

**Figure 2:**
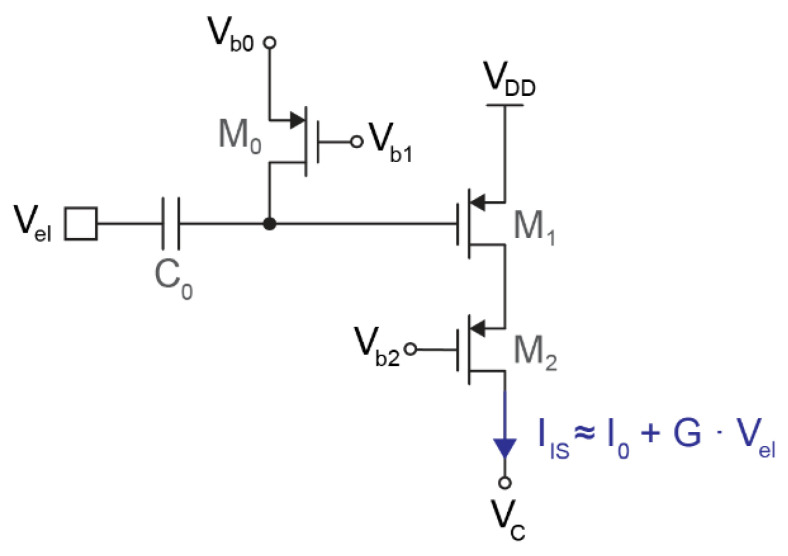
Schematic of the input stage, consisting of a high-pass filter (C_0_ and M_0_), a single PMOS transistor operating as transconductor (M_1_), and a cascode (M_2_).

Figure 2 shows the schematic of the input stage, which consists of a high-pass filter followed by a single-ended transconductor. The high-pass filter is based on a metal–insulator–metal (MIM) capacitor (C_0_) and a PMOS pseudo-resistor (M_0_). C_0_ must be as large as possible since this capacitor forms a voltage divider with the input capacitance of the next stage (M_1_). However, given the relatively large size of MIM capacitors, the value of C_0_ is limited to either hundreds of femtofarads or a few picofarads, depending on the application. The modulator implemented can be programmed with either C_0_ = 4.25 pF (for full-band low-noise applications) or C_0_ = 350 fF (for compact action–potential readout).

**Figure 3:**
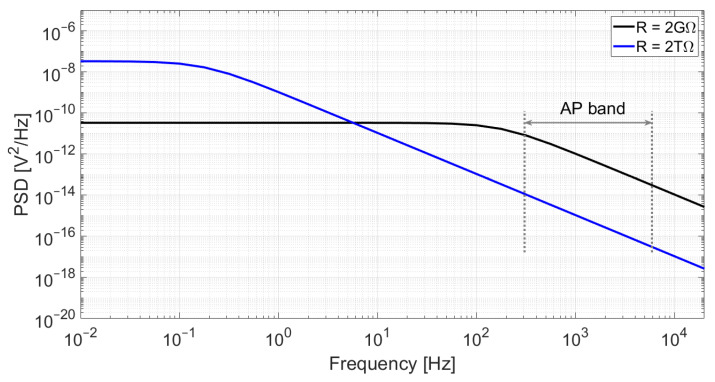
Simulation of the output noise of an RC low-pass filter for C = 350 fF and two different resistor values: R = 2 GΩ (**black**) and R = 2 TΩ (**blue**). Although a higher resistance value yields higher power spectral density at low frequencies, the cut-off frequency is significantly reduced, which results in lower noise at high frequencies. In this example, the integrated noise in the action-potential band (300 Hz–6 kHz) is 58 μVrms for R = 2 GΩ and only 2.0 μVrms for R = 2 TΩ.

Given the small input capacitance, a very high-ohmic pseudo-resistor is needed to set the cut-off frequency of the high-pass filter well below 1 Hz. These filter characteristics are required to avoid signal attenuation or phase shifts in the band of interest but also to reduce the effect of the thermal noise generated by M_0_ since, as shown in Figure 3, the noise is low-pass-filtered by the RC circuit. As a consequence, the pseudo-resistor was tuned in the TΩ range.

The transconductor is based on a single PMOS transistor (M_1_) in weak inversion. The main transistor is complemented with a cascode (M_2_) to increase the output impedance and keep the output current independent from the output voltage V_C_, which can oscillate up to ±200 mV around 900 mV. Assuming small input voltages, the current generated by this transconductor follows

(1) $$I_{\mathrm{IS}}{=I}_{0}{+G\cdot V}_{\mathrm{el}},$$

where I_0_ is the DC biasing current, and G is the transconductance of M_1_ multiplied by the attenuation due to the capacitive voltage divider. The biasing current I_0_ chosen for all the measurements reported in this article is 1.4 μA, although the integrated circuit allows for tuning this current in the [200 nA, 1.6 μA] range to define different levels of power consumption and noise.

Two variants of this circuit have been implemented in the prototype chip: one optimized for compact neural interfaces and a second variant optimized for low noise. The compact transconductor is coupled to the 350 fF input capacitor and consists of M_1_ = 30 μm/1.2 μm and M_2_ = 10 μm/1.2 μm. The voltage divider, formed by the input capacitance (350 fF) and the gate capacitance of M_1_ (95 fF), attenuates the input signal by 2 dB and prevents the use of a larger transistor. The resulting transconductance of the input stage is G ≈ 20 V^−1^ · I_0_. The alternative low-noise transconductor is coupled to the 4.25 pF input transistor to avoid any signal attenuation in the capacitive voltage divider. Therefore, the size of M_1_ was increased up to 70 μm/1.5 μm (with M_2_ = 10 μm/1.5 μm), which reduces flicker noise at low frequencies. The resulting transconductance is G ≈ 25 V^−1^ · I_0_.

Although this transconductor topology is sensitive to power-supply noise and process variations, inverts the input signal, and is inherently nonlinear, it can be very compact and potentially feature low noise. Process variations can cause minor gain variations, which can be coped with through calibration. Nonlinearity may cause the distortion of very large input signals, but action potentials are expected to be smaller than 1 mV. The power-supply rejection ratio (PSRR) is nearly 0 dB since the output current directly depends on the V_SG_ of M_1_ and, therefore, on the supply voltage. Low-noise external voltage regulators are required to minimize the power-supply noise, and data post-processing can be included to attenuate power-supply noise (50/60 Hz harmonics) and other types of predictable noise. Note that in the case of a MEA with multiple copies of the same converter, the power-supply noise is common to all of them, which makes the extraction and subtraction of common noise during data post-processing possible. The feasibility and robustness of compact, single-ended input stages for MEAs have also been recently proven in [12].

### 2.2. Feedback IDAC

**Figure 4:**
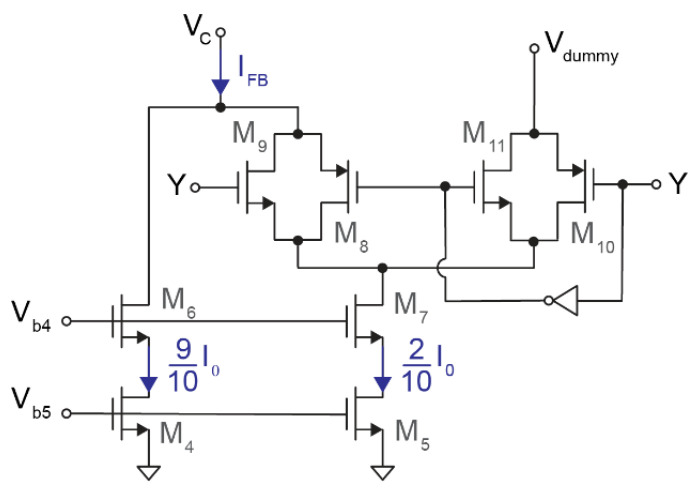
Schematic of the 1-bit feedback IDAC. A first current source (M_4_) provides 0.9·I_0_ to the output, while the current generated by a second current source (M_5_) is injected into the output or discarded depending on the state of Y.

The current DAC consists of two current sources and two transmission gates. As shown in Figure 4, M_4_ and M_6_ generate I_M4_ = 0.9·I_0_ while M_5_ and M_7_ produce I_M5_ = 0.2·I_0_. Therefore, the instantaneous output current is

(2) $$I_{\mathrm{FB}}{=I}_{0}\cdot (0.9+0.2\cdot Y),$$

given that—depending on the feedback signal Y—transmission gates M_8_–M_11_ control whether the current I_M5_ is connected to V_C_, the output, through M_8_–M_9_ or discarded via M_10_–M_11_. As for the input stage, the biasing current I_0_ was fixed at 1.4 μA.

The modulator is stable only if any possible I_IS_ current ranges between the two possible feedback currents. According to Equations (1) and (2), this condition is met if $\left|V_{\mathrm{el}}\right|$ < 5 mV for C_0_ = 350 fF (G ≈ 20 V^−1^ · I_0_) and if $\left|V_{\mathrm{el}}\right|$ < 4 mV for C_0_ = 4.25 pF (G ≈ 20 V^−1^ · I_0_). Nevertheless, the practically available full scale is considered to be 3 mV_p_ in order to limit distortion and to avoid saturation and excessive quantization noise [22,23]. Moreover, this full-scale reduction relaxes requirements in terms of matching and robustness against process variations, since deviations from nominal parameters would not saturate the converter and could be corrected during digital-signal post-processing.

### 2.3. Integrator

The difference between input and feedback currents, I_IS_–I_FB_, flows through capacitor C_int_, which acts as an integrator. The value of this capacitor defines the integration constant and, along with the biasing current and the voltage-controlled oscillator (VCO)-quantizer gain, the modulator transfer functions. The nominal capacitance for I_0_ = 1.4 μA is C_int_ = 775 fF, but a 5-bit programmable capacitor has been implemented to allow for capacitances from 25 fF to 775 fF in order to accommodate different biasing currents without significant changes in the state variable V_C_.

### 2.4. VCO-Based Quantizer

A VCO-based quantizer has been used to achieve second-order noise shaping without the need for a second analog integrator. The quantizer is the combination of a VCO whose frequency is modulated by voltage V_C_ and a frequency-to-digital (F2D) converter whose output is a logic ‘1’ when a pulse from the VCO is detected during the preceding sampling period. A VCO-based quantizer can be modelled as a frequency integrator (the instantaneous VCO phase is the result of integrating the VCO frequency over time), followed by a phase quantizer and a discrete-time derivative [20,21]. The spectral properties of the resulting signal can be analyzed by modelling the VCO-based quantizer as a pulse–frequency modulator (PFM) [23].

**Figure 5:**
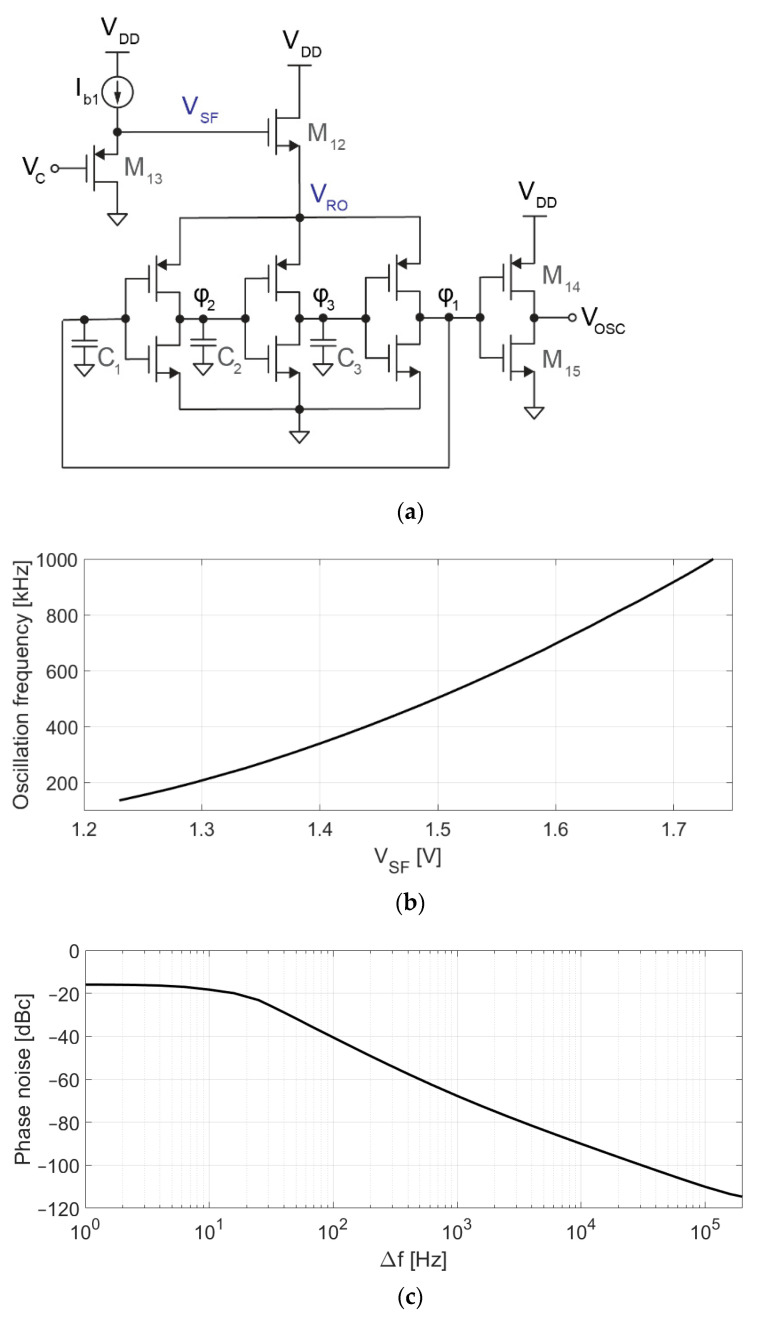
Voltage-controlled oscillator. (**a**) Schematic of the VCO, consisting of a 3-stage ring oscillator, a level shifter (M_14_–M_15_), a source follower driving the ring oscillator (M_12_), and a second source follower (M_13_) for adapting the DC voltage level from V_C_ (0.9 V) to V_SF_ (1.5 V); (**b**) Simulated voltage-to-frequency response; (**c**) Simulated phase noise.

Figure 5a shows the schematic of the VCO. The core of this circuit is a 3-stage voltage-controlled ring oscillator, whose frequency depends on V_SF_, as shown in Figure 5b. For V_SF_ = 1.5 V, the oscillation frequency and gain are f_VCO_ = 500 kHz and K_VCO_ = 1.6 kHz/mV with a current consumption of 250 nA. Transistor dimensions are 5 μm/6 μm for PMOS and 2 μm/6 μm for NMOS. Capacitors C_1_–C_3_ (70 fF) have been used to reduce the oscillation frequency, which would otherwise be too high or require too low of a current to bias M_12_. V_RO_ is controlled by M_12_ (400 nm/20 μm), which acts as a source follower using the oscillator current for its own biasing. The gate of M_12_ cannot be directly driven by V_C_ since the target $\bar{V_{C}}$ (0.9 V) is lower than the target $\bar{V_{\mathrm{SF}}}$ (1.5 V). M_13_ is a second source follower, used to adapt the DC level of V_C_ to the 1.5 V required at V_SF_ to set the oscillation frequency to around 500 kHz. Finally, M_14_ and M_15_ act as a level shifter, adapting the 620 mV_pp_ oscillation at *φ*_1_ to the rail-to-rail levels demanded by digital circuitry.

The proposed VCO is sensitive to process variations, and the exact relationship between the input voltage and the output frequency is difficult to predict. Fortunately, since the VCO operates in a closed-loop system, any deviation from the nominal behavior (e.g., the VCO being slower than expected at V_C_ = 0.9 V) would be compensated by the loop (e.g., higher $\bar{V_{C}}$, correcting the average oscillation frequency). The VCO was optimized to minimize the impact of phase noise (shown in Figure 5c) and distortion in the performance of the converter [24], which is also mitigated by the closed-loop architecture. Time-domain simulations were used to verify that the performance of the modulator is not limited by phase noise or VCO distortion.

**Figure 6:**
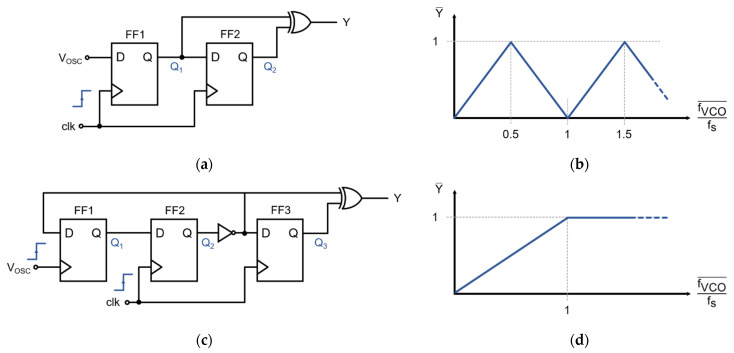
Frequency-to-digital (F2D) converters. (**a**) Classical 2-flip-flop exclusive OR (XOR)-based frequency-to-digital converter; (**b**) Normalized frequency-to-digital response of the classical XOR-based F2D, which is not monotonic and may feature metastable states in the closed-loop converter; (**c**) Proposed frequency-to-digital converter, with a third flip-flop to force the saturation of the F2D; (**d**) Normalized frequency-to-digital response of the proposed F2D, which is monotonic and prevents metastable operating points.

A frequency-to-digital converter is required to transform the asynchronous VCO oscillation into a synchronous pulse–frequency-modulated signal [21,23]. Figure 6a shows a classical F2D converter circuit that is based on two D-type flip-flops (FF) and an XOR-gate. Ideally, the output of this F2D is a logic ‘1’ if a VCO transition—either rising or falling—has been registered during the last sampling period. However, when transitions occur faster than the sampling frequency (i.e., f_VCO_ > 0.5·f_s_), a fraction of the transitions is missed during sampling, and the frequency of the output pulses decreases for faster VCO frequencies. Figure 6b shows that the average output is a function of the normalized VCO frequency and that it is periodic. This F2D converter topology is frequently used in other modulator architectures for which the oscillation frequency is guaranteed to be lower than 0.5·f_s_. However, the closed-loop modulator presented in this work is intended to operate around $\bar{f_{\mathrm{VCO}}}$ ≈ 0.5·f_s_. The F2D converter of Figure 6a would not be suitable for this application since the modulator could find undesirable metastable operation points at higher oscillation frequencies, especially at $\bar{f_{\mathrm{VCO}}}$ ≈ 1.5·f_s_.

The F2D converter used in this design is a variation of the classical exclusive OR (XOR)-based approach, depicted in Figure 6c. When the oscillation frequency is slower than the sampling frequency (i.e., for f_VCO_ < f_s_), each pulse at V_OSC_ toggles FF1, and this change is then registered by FF2, producing a logic ‘1’ at the output of the converter. When no pulses are received during a sampling period, Q_3_ = Q_2_, which renders Y = 0. For f_VCO_ > fs, the F2D converter saturates, and the output is constantly ‘1’ since FF1 would toggle once every sampling period. This saturation at high frequencies, illustrated in Figure 6d, improves the stability of the system since only a specific range of frequencies around f_s_/2 is possible during normal operation.

## 3. Electrical Characterization

**Figure 7:**
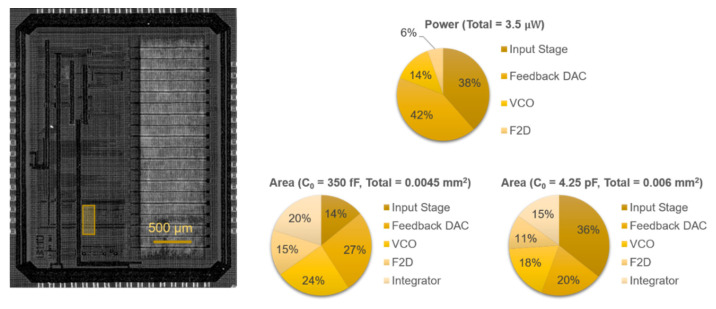
Chip micrograph of the prototype fabricated in 0.18 μm CMOS, with the location of the system highlighted, and area and simulated power breakdowns.

The proposed readout circuit has been prototyped in 0.18-μm CMOS technology (1P6M). Figure 7 shows the 3 × 3.8 mm^2^ chip, on which the highlighted 130 × 330 μm^2^ area was used for testing different ∆Σ configurations. Excluding the biasing and auxiliary circuitry required for testing, the building blocks of the compact modulator (C_0_ = 350 fF) occupied 0.0045 mm^2^, while the low-noise modulator occupied 0.006 mm^2^ due to the larger capacitor (C_0_ = 4.25 pF).

**Figure 8:**
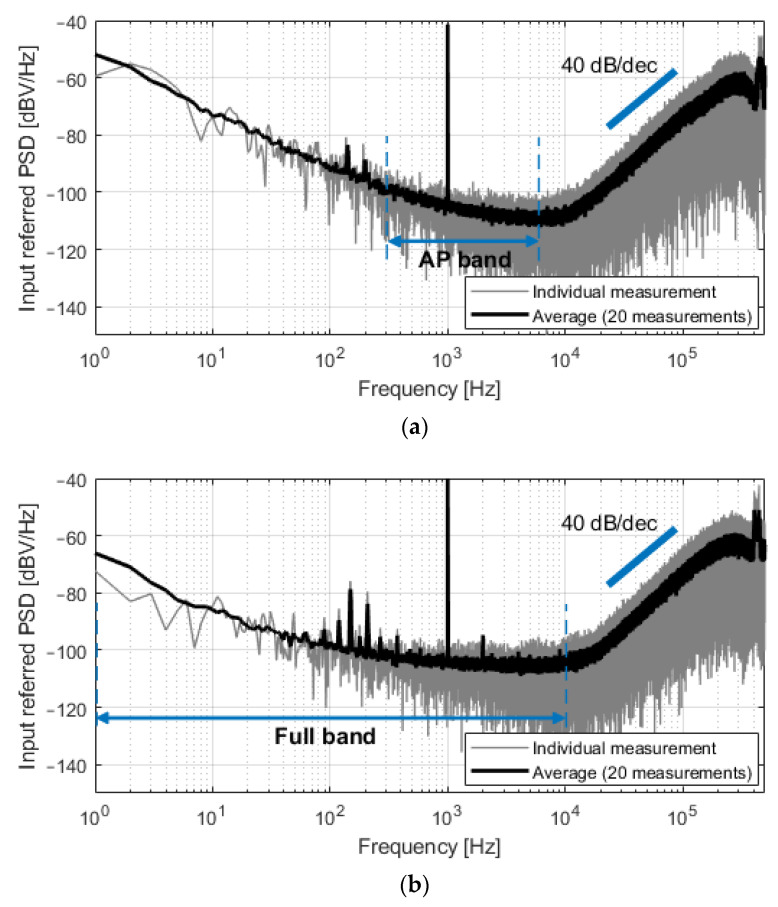
Spectra of the measured output bitstreams, referred at the input of the converter, for a sinusoidal input of 200 μV_p_ at 1 kHz. (**a**) For C_0_ = 350 fF, the input-referred noise in the 300 Hz–6 kHz band was 5.0 μV_rms_; (**b**) For C_0_ = 4.25 pF, the input-referred noise in the 1 Hz–10 kHz band was 8.7 μV_rms_.

The system was first characterized by applying a 200 μV_p_ sinusoidal input signal at 1 kHz. Figure 8 shows the spectra of the output bitstreams for both C_0_ = 350 fF and C_0_ = 4.25 pF. The spectrum in gray is the result of single measurements, while the black plot represents the average magnitude of 20 consecutive measurements. Second-order noise shaping is visible at high frequencies, and the input-referred noise was 5.0 μV_rms_ in the 300 Hz–6 kHz band (C_0_ = 350 fF, Figure 8a) and 8.7 μV_rms_ in the 1 Hz–10 kHz band (C_0_ = 4.25 pF, Figure 8b). Unexpected noise is present in the 80–500 Hz band and is especially visible in Figure 8b due to lower flicker noise. This noise is attributed to the measurement setup; however, its contribution to the total integrated in-band noise is minor.

**Figure 9:**
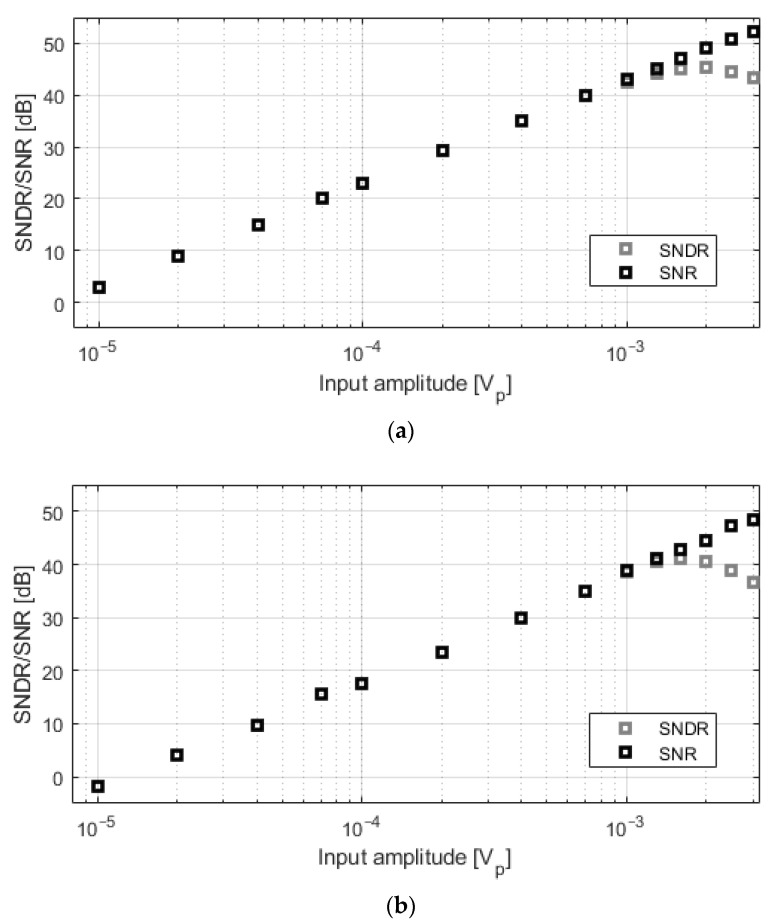
Measured SNDR and SNR for different input amplitudes at 1 kHz. (**a**) C_0_ = 350 fF; (**b**) C_0_ = 4.25 pF.

Figure 9 depicts the signal-to-noise ratio (SNR) and the signal-to-noise-and-distortion ratio (SNDR) for different input amplitudes at 1 kHz. Each point represents the average of 20 consecutive measurements. Signals larger than 1–1.5 mV_p_ are limited by the distortion of the input-stage transconductor.

The performance of the proposed readout is summarized in Table 1 and compared to the prior art, including readout circuits integrated into arrays for in vitro [3,9,11,12] platforms, in vivo implants [5,10,15,17], and standalone converters [13,16]. Our work features low noise characteristics (<6 μV_rms_), low power consumption (<5 μW/ch), and a compact footprint (<0.01 mm^2^/ch), which is in line with the state of the art. The overall performance of the converter presented in [13] appears superior; however, the converter reported here was implemented in 0.18-μm CMOS and provided synchronous output, which may be advantageous depending on the application. Finally, it is noteworthy that the converter reported in [17] achieves comparable metrics while it includes on-chip decimation filters.

## 4. In Vitro Validation

**Figure 10:**
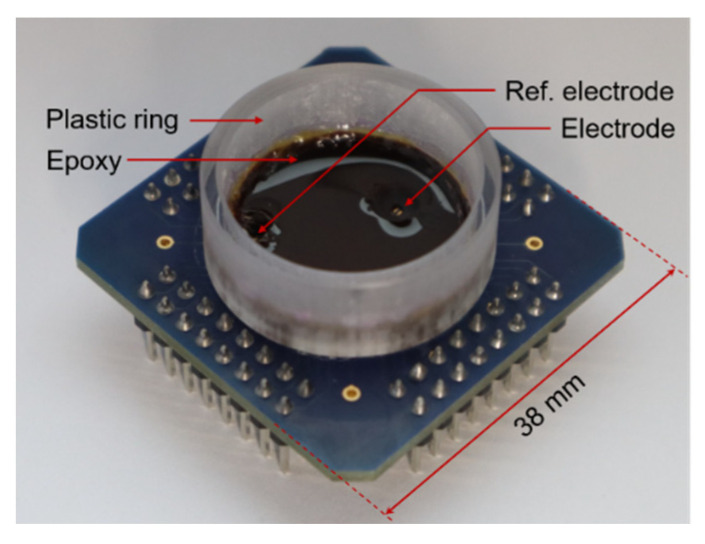
Chip and package used for in vitro validation. The plastic ring defines the well by which cells and cell culture medium are confined during recordings. The bottom of the well is mainly covered with epoxy, with the exception of the electrode area.

As a proof of concept, the implemented modulator was used to sense the field potentials of cardiac tissue. The CMOS prototype was wire-bonded to a custom-made PCB, where a 0.24 × 1.54 mm^2^ pad, covered with electroless nickel immersion gold (ENIG), was used as the electrode. Another ENIG electrode was used as a reference electrode and was connected to V_DD_ = 1.8 V. As shown in Figure 10, a plastic ring was glued to the PCB, and the ASIC and bonding wires were covered with epoxy to leave only the selected electrodes exposed. These materials were selected in order to simplify the fabrication process and were stable enough for system characterization measurements but would not be suitable for long-term recordings.

The chip was cleaned and sterilized, and the electrodes were coated with human fibronectin to facilitate cell attachment. Human induced pluripotent stem cells (hIPSCs) derived from a healthy donor were purchased from FUJIFILM Cellular Dynamics Inc. The human induced pluripotent stem cell (hIPSC) line, CW30318CC1, was obtained from the CIRM hPSC Repository funded by the California Institute of Regenerative Medicine (CIRM). The hIPSCs were then differentiated into spontaneously and synchronously beating cardiomyocytes. The cardiomyocytes were carefully lifted from the cell culture dish to not disrupt the cell-cell connections. The cardiomyocyte tissue was then transferred and allowed to adhere to the surface with the coated electrodes. 

**Figure 11:**
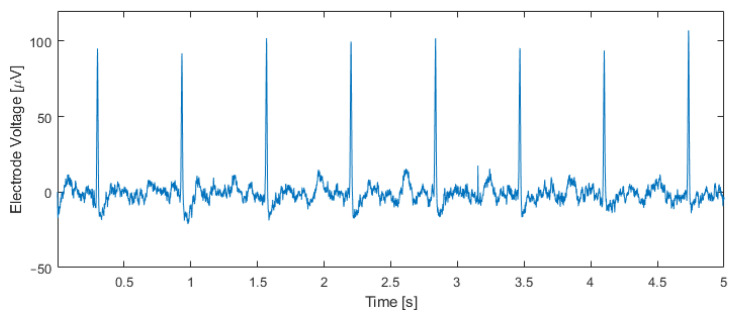
Measurement results using human IPSC-derived cardiomyocytes. The output bitstream of the modulator (C_0_ = 4.25 pF) was band-pass filtered between 5 Hz and 200 Hz, unveiling cardiac potentials and a beating rate of 95 beats per minute, which matches the beating rate measured optically.

Figure 11 shows an electrical recording taken inside an incubator one hour after transferring the tissue to the chip. The input capacitor was set to C_0_ = 4.25 pF, and the output bitstream was band-pass filtered with a fourth-order Butterworth band-pass filter between 5 Hz and 200 Hz. Cardiac action potentials with an amplitude of approximately 100 μV_pp_ and a beating rate of 95 beats per minute are visible. This beating rate matches the rate measured by observing the contractions of the tissue through the microscope.

## 5. Conclusions

This paper proposes a readout circuit for bioelectrical signals based on a ∆Σ modulator with a VCO-based quantizer, which achieves second-order noise shaping with minimal analog circuitry. The size of the input capacitor plays a fundamental role in the design, as it defines the signal attenuation and the size of the input stage. A large P-type transistor serves as the input transconductor, which minimizes flicker noise without the need for chopping. Another capacitor was used for the integration of both input and feedback currents, eliminating the need for operational amplifiers. A novel frequency-to-digital converter was developed to improve the stability of the VCO-based quantization. The performance of the converter matches that of state-of-the-art devices in terms of noise, power, and area and constitutes a competitive solution for extracellular action–potential detection in large-scale electrode arrays and neural interfaces.

The resulting converter architecture is very simple and avoids conventional problems of more complex modulators, such as feedback DAC non-linearity. However, the proposed circuit is not inherently robust against power-supply noise, process variations, or input-stage non-linearity. Therefore, the modulator relies on low-noise external voltage regulators to minimize power-supply noise, and off-chip digital filtering can be used to attenuate noise at specific frequencies. Process variations may result in unexpected gain deviations, making calibration necessary if the absolute amplitude of signals is of interest. The non-linearity of the input stage can be neglected for small input signals, and distortion–compensation methods should be explored if large input signals are expected.

## Figures and Tables

**Table 1:** Performance summary and comparison with state-of-the-art.

	[3]	[13]	[10]	[5]	[11]	[9]	[15]	[16]	[17]	[12]	This Work	
											C_0_ = 4.25 pF	C_0_ = 350 fF
**Year**	2017	2018	2018	2018	2018	2020	2020	2021	2021	2021	**2021**	
**Architecture**	SAR	ATC	SAR	∆-∆Σ	SAR	SS	∆Σ (VCO-Q)	∆Σ	I∆Σ	SAR	**∆Σ** **(VCO-Q)**	
**Technology** **(nm)**	180	65	250	180	130	90/65	110	130	180	180	**180**	
**Sampling frequency** **(Hz)**	20 k	-^e^	31.25 k	25 k	30 k	70 k	1.28 M	10 M	20 k	11.6 k	**1M**	
**Bandwidth** **(Hz)**	300–10 k	11 k	10–10 k	0.5–12.7 k	300–10 k	300–10 k	10 k	1–500	300–10 k	300–5 k	**1–10 k**	**300–6 k**
**Area/channel** **(mm^2^/ch)**	0.024 ^a^	0.006	0.155	0.058 ^c^	0.043 ^a^	0.014 ^a^	0.078	0.011 ^c^	0.0046 ^c^	0.001 ^a^	**0.006 ^b^**	**0.0045** ^**b**^
**Power/channel** **(μW/ch)**	16	1.2	2.5	3.05	46	130	6.5	0.99 ^c^	8.59 ^c^	5.9	**3.5** ^**b**^	
**Input-referred noise** **(** **μ** **V_rms_)**	2.4	3.8	5.62	3.32	7.5	5.5	9.5	2.6	4.37	10.4	**8.7**	**5.0**
**Input range** **(mV_pp_)**	-	4	-	-	-	-	300	10 ^d^	14	8	**6**	

SAR: Successive Approximation Register; ATC: Analog-to-Time Converter; SS: Single-slope; I∆Σ: Incremental ∆Σ; VCO-Q: VCO-based quantizer. ^a^ Estimated. ^b^ Excluding biasing. ^c^ Including on-chip digital filter. ^d^ Scalable with the sampling frequency. ^e^ Asynchronous output.

## References

[1] Hierlemann A., Frey U., Hafizovic S., Heer F. (2011). Growing cells atop microelectronic chips: Interfacing electrogenic cells in vitro with CMOS-based microelectrode arrays. Proc. IEEE.

[2] Musk E., Neuralink (2019). An integrated brain-machine interface platform with thousands of channels. J. Med. Internet Res..

[3] Dragas J., Viswam V., Shadmani A., Chen Y., Bounik R., Stettler A., Radivojevic M., Geissler S., Obien M.E.J., Müller J. (2017). In Vitro Multi-Functional Microelectrode Array Featuring 59760 Electrodes, 2048 Electrophysiology Channels, Stimulation, Impedance Measurement, and Neurotransmitter Detection Channels. IEEE J. Solid-State Circuits.

[4] Kollo M., Racz R., Hanna M.E., Obaid A., Angle M.R., Wray W., Kong Y., Müller J., Hierlemann A., Melosh N.A. (2020). CHIME: CMOS-Hosted in vivo Microelectrodes for Massively Scalable Neuronal Recordings. Front. Neurosci..

[5] Park S.Y., Cho J., Na K., Yoon E. (2018). Modular 128-Channel Δ—ΔΣ Analog Front-End Architecture Using Spectrum Equalization Scheme for 1024-Channel 3-D Neural Recording Microsystems. IEEE J. Solid-State Circuits.

[6] Najafi K., Wise K.D. (1986). An Implantable Multielectrode Array with On-Chip Signal Processing. IEEE J. Solid-State Circuits.

[7] Mora Lopez C., Putzeys J., Raducanu B.C., Ballini M., Wang S., Andrei A., Rochus V., Vandebriel R., Severi S., Van Hoof C. (2017). A Neural Probe with Up to 966 Electrodes and Up to 384 Configurable Channels in 0.13 μm SOI CMOS. IEEE Trans. Biomed. Circuits Syst..

[8] Rey H.G., Pedreira C., Quian Quiroga R. (2015). Past, present and future of spike sorting techniques. Brain Res. Bull..

[9] Kato Y., Matoba Y., Honda K., Ogawa K., Shimizu K., Maehara M., Fujiwara A., Odawara A., Yamane C., Kimizuka N. High-Density and Large-Scale MEA System Featuring 236, 880 Electrodes at 11.72 μm Pitch for Neuronal Network Analysis. Proceedings of the 2020 IEEE Symposium on VLSI Circuits.

[10] Chang S., Park S.Y., Yoon E. (2018). Minimally-invasive neural interface for distributed wireless electrocorticogram recording systems. Sensors.

[11] Lopez C.M., Chun H.S., Wang S., Berti L., Putzeys J., Van Den Bulcke C., Weijers J.W., Firrincieli A., Reumers V., Braeken D. (2018). A multimodal CMOS MEA for high-throughput intracellular action potential measurements and impedance spectroscopy in drug-screening applications. IEEE J. Solid-State Circuits.

[12] Yuan X., Hierlemann A., Frey U. (2021). Extracellular Recording of Entire Neural Networks Using a Dual-Mode Microelectrode Array with 19 584 Electrodes and High SNR. IEEE J. Solid-State Circuits.

[13] Leene L.B., Constandinou T.G. (2018). A 0.006 mm^2^ 1.2 μ W Analog-to-Time Converter for Asynchronous Bio-Sensors. IEEE J. Solid-State Circuits.

[14] Ballini M., Muller J., Livi P., Chen Y., Frey U., Stettler A., Shadmani A., Viswam V., Lloyd Jones I., Jackel D. (2014). A 1024-Channel CMOS Microelectrode Array with 26,400 Electrodes for Recording and Stimulation of Electrogenic Cells In Vitro. IEEE J. Solid-State Circuits.

[15] Lee C., Jeon T., Jang M., Park S., Kim J., Lim J., Ahn J., Huh Y., Chae Y. (2020). A 6.5-μW 10-kHz BW 80.4-dB SNDR Gm C-Based CT Modulator with a Feedback-Assisted Gm Linearization for Artifact-Tolerant. IEEE J. Solid-State Circuits.

[16] Pazhouhandeh M.R., Kassiri H., Shoukry A., Weisspapir I., Carlen P.L., Genov R. (2021). Opamp-Less Sub-μW/Channel Delta-Modulated Neural-ADC with Super-G Input Impedance. IEEE J. Solid-State Circuits.

[17] Wendler D., De Dorigo D., Amayreh M., Bleitner A., Marx M., Manoli Y. A 0.00378 mm^2^ Scalable Neural Recording Front-End for Fully Immersible Neural Probes Based on a Two-Step Incremental Delta-Sigma Converter with Extended Counting and Hardware Reuse. Proceedings of the 2021 IEEE International Solid-State Circuits Conference (ISSCC).

[18] Schreier R., Temes G.C. (2005). Understanding Delta-Sigma Data Converters.

[19] Park M., Perrott M.H. (2009). A 78 dB SNDR 87 mW 20 MHz Bandwidth Continuous-Time DS ADC with VCO-Based Integrator and Quantizer Implemented in 0.13 μm CMOS. IEEE J. Solid-State Circuits.

[20] Straayer M.Z., Perrott M.H. (2008). A 12-Bit, 10-MHz Bandwidth, Continuous-Time SD ADC with a 5-Bit, 950-MS/s VCO-Based Quantizer. IEEE J. Solid-State Circuits.

[21] Kim J., Jang T.K., Yoon Y.G., Cho S.H. (2010). Analysis and design of voltage-controlled oscillator based analog-to-digital converter. IEEE Trans. Circuits Syst. I Regul. Pap..

[22] Ardalan S.H., Paulos J.J. (1987). An Analysis of Nonlinear Behavior in Delta-Sigma Modulators. IEEE Trans. Cir. Syst..

[23] Gutierrez E., Hernandez L., Cardes F., Rombouts P. (2018). A Pulse Frequency Modulation Interpretation of VCOs Enabling VCO-ADC Architectures with Extended Noise Shaping. IEEE Trans. Circuits Syst. I Regul. Pap..

[24] Cardes F., Quintero A., Gutierrez E., Buffa C., Wiesbauer A., Hernandez L. (2018). SNDR limits of oscillator-based sensor readout circuits. Sensors.

